# An Analytical Treatise on Digestion, Considered Especially in Man and in Vertebrated Animals

**Published:** 1844-10

**Authors:** 


Art. V.—*Traité Analytique de la Digestion; considérée particulièrement dans l'Homme et dans les Animaux vertébrés.* Par N. Blondlot, Docteur en Médecine, Professeur (adjoint) de Chimie à l'Ecole de Médecine de Nancy, &c. &c.—*Paris*, 1843.
*An Analytical Treatise on Digestion, considered especially in Man and in Vertebrated Animals.* By N. Blondlot, Doctor of Medicine.—*Paris*, 1843. 8vo, pp. 472.

This treatise contains the results of a series of experiments, which were made with the view of following out the line of inquiry that had been so successfully opened by Dr. Beaumont. As cases of fistulous opening in the stomach, however, do not present themselves every day in the human subject,—still less frequently, in a subject possessing the vigorous health of Alexis St. Martin,—Dr. Blondlot proceeded to *establish* them in a couple of unfortunate dogs, by means of incisions through the abdomidal parietes; and he states that one of them is still alive, and in excellent condition, the other having been sacrificed for a particular object. The results which he has obtained do not appear to us sufficiently important to require more than a brief notice. He maintains that the salivary fluid has no influence in accelerating the process of digestion;—a position which is probably far too exclusive. We can as little accord with the conclusions of Dr. Wright, who (in a series of papers communicated to the Lancet not long since) maintains that the saliva is *the essential* agent in the digestive process. We believe that the truth, as usual, lies between the two extremes; and that the peculiar matter of the saliva acts as an incipient *ferment*, especially upon the farinaceous part of the food. The ready solubility of the animal tissues in the gastric fluid, probably renders the preliminary process of insalivation less important in the carnivorous tribes than in the herbivorous.

M. Blondlot's chemical analyses of the gastric fluid of the dog have led him to the remarkable conclusion, that neither the hydrochloric nor the acetic acid exists in it in a free state; and that its acidity is due to the presence of the acid phosphates of lime and ammonia. He founds the former part of this statement upon the fact, that neither of these acids could be obtained from the fluid by distillation. But that this conclusion is not generally applicable, is evident from the fact that, *by the very same method*, Dr. Dunglison obtained *a large quantity* of these acids, especially the former, from the gastric fluid of Alexis St. Martin. M. Blondlot considers the animal matter in the gastric fluid in the light of a *ferment;* in which he accords with the results of the most recent chemical inquiries. He regards starchy matters as properly *dissolved* in the gastric fluid, provided the starch-vesicles are ruptured; whilst protein-compounds, gelatin, and vegetable tissues are only *reduced* to a molecular pulp. We scarcely think that this division will stand the test of further inquiry.

